# Efficacy of Multisensory Technology in Post-Stroke Cognitive Rehabilitation: A Systematic Review

**DOI:** 10.3390/jcm11216324

**Published:** 2022-10-26

**Authors:** Alessandra Parisi, Francesca Bellinzona, Daniele Di Lernia, Claudia Repetto, Stefano De Gaspari, Giulia Brizzi, Giuseppe Riva, Cosimo Tuena

**Affiliations:** 1 Department of Psychology, Università Cattolica del Sacro Cuore, Largo Gemelli, 1, 20121 Milan, Italy; 2 Humane Technology Laboratory, Università Cattolica del Sacro Cuore, Largo Gemelli, 1, 20121 Milan, Italy; 3 Applied Technology for Neuro-Psychology Laboratory, IRCCS Istituto Auxologico Italiano, Via Magnasco 2, 20149 Milan, Italy

**Keywords:** post-stroke dementia, embodiment, virtual reality, multisensory integration

## Abstract

Post-stroke, in addition to sensorimotor signs and symptoms, could lead to cognitive deficits. Theories of embodiment stress the role of sensorimotor system and multisensory integration in sustaining high-order cognitive domains. Despite conventional post-stroke cognitive rehabilitation being effective, innovative technologies could overcome some limitations of standard interventions and exploit bodily information during cognitive rehabilitation. This systematic review aims to investigate whether ‘multisensory technologies’ compared to usual care treatment can be a viable alternative for cognitive rehabilitation. By applying PRISMA guidelines, we extracted data and assessed the bias of 10 studies that met the required criteria. We found that multisensory technologies were at least comparable to standard treatment but particularly effective for attention, spatial cognition, global cognition, and memory. Multisensory technologies consisted principally of virtual reality alone or combined with a motion tracking system. Multisensory technologies without motion tracking were more effective than standard procedures, whereas those with motion tracking showed balanced results for the two treatments. Limitations of the included studies regarded the population (e.g., no study on acute stroke), assessment (e.g., lack of multimodal/multisensory pre-post evaluation), and methodology (e.g., sample size, blinding bias). Recent advancements in technological development and metaverse open new opportunities to design embodied rehabilitative programs.

## 1. Introduction

Stroke is one of the main causes of death and disability in the world [1] and its effects can include, in addition to sensory and motor impairments, cognitive deficits that can have a strong negative impact on quality of life [2].

Cognitive deficits comprise a wide spectrum of manifestations that vary depending on the staging (i.e., acute/subacute or chronic), number (i.e., single stroke or multiple cerebrovascular accidents), etiology (ischemic or hemorrhagic), and region (cortical, subcortical, brainstem/cerebellum) of the stroke(s). In the acute phase (i.e., the first hours/week after the cerebrovascular accident) of a stroke, the most prevalent disorders are those affecting executive functioning (39%) and visual perception/construction (38%) [3]. According to previous research [3], it is possible to establish an association between the brain hemisphere affected by the stroke and the following impairment. For example, left lesions to seem to affect predominantly language, abstract reasoning, verbal memory, and executive functions, whereas right strokes have been reliably demonstrated to be associated with visuospatial neglect; instead, other functions such as visual memory and visual perception/construction performance seem to be similar between left- and right-hemisphere strokes [3]. Interestingly, subcortical stroke does not have this clear-cut distinction in cognitive deficits [3]. Lastly, cerebellar stroke could result in the so-called cerebellar cognitive and affective syndrome (involving executive, visuospatial, and language functions and personality) [4]. In the chronic phase, cognitive impairment can be observed, and, if a progressive cognitive deterioration is present (usually after six months from the accident), a variant of vascular dementia called post-stroke dementia might be diagnosed [5]. Neuropsychological assessments conducted in this phase show deficits in attention, mental processing speed, executive functioning, and visual perception [6,7,8]. However, neuropsychological profiles that occur in vascular dementia are heterogeneous and depend on the number, side, and location(s) of the lesion(s). Lastly, the cognitive outcome of acute and chronic stroke depends also on the etiology, where hemorrhagic strokes could result in greater impairment [3,9,10].

In addition to these problems, it has been noticed that stroke could lead to other dysfunctions. Studies have shown that stroke could result in disturbances in multisensory integration (MSI) processes, which correspond to the integration of information from different sensory modalities into a unique percept. Indeed, a potential consequence of stroke in the acute phase is the alteration of body representation due to the altered integration of sensorimotor bodily information [11]. A study on chronic stroke patients found that MSI was impaired in left-hemisphere but not in right-hemisphere stroke patients [12]. Again, MSI deficits could vary depending on the stroke lesion(s) and region. Indeed, a widespread network of parietal, temporal, frontal, and primary cortices is thought to sustain MSI processes [13].

MSI mechanisms are crucial for humans [14,15,16,17] as they play a critical role in bodily self-consciousness and interaction with the environment. As a result, brain lesions affecting MSI processing can cause various problems such as feeling hyperactivated by sensory stimuli or having difficulty effectively integrating different sensory information (e.g., visual and auditory). For example, to move hands accurately, information from different sensory systems must be combined (e.g., visual, proprioceptive, and tactile systems) [18]. Originally, MSI was thought to be a process primarily controlled by bottom-up functions, but several studies have found that the multisensory integration process is also strongly linked to top-down processes, involving memory and attention [19,20]. In addition, recent findings in the field of embodied cognition suggest that sensorimotor/bodily information is crucial to create a multimodal cognitive representation of objects, events, affective experiences, and the environment [21,22,23]. According to this approach [21], humans create sensorimotor simulations when perceiving an object or representing an event/experience. Later, when the object (e.g., an event) of the experience is re-experienced, a multimodal representation of it arises as it was encoded. Given the crucial role of sensorimotor information and MSI for cognition and because such aspects can be affected in post-stroke patients, there is a clear need to provide a cognitive rehabilitation that also integrates cognitive operations with bodily and sensorimotor information [24].

Currently, cognitive rehabilitation in post-stroke patients provides a conventional treatment that includes paper-and-pencil exercises [25] and computer-based tasks [26], which include computer exercises and games specifically designed to improve cognitive functions such as memory and attention.

Despite this, conventional rehabilitation is still a widely used method due to its accessibility, as well as ease of use, clinical validation, and low cost [27]. On the other hand, computerized rehabilitation offers exercises similar to paper-pencil ones in which the patient interacts with a computer equipped with a screen and keyboard. This type of rehabilitation intervention seemed to have a significant positive effect, especially for visuospatial deficits [28], and it also appeals to more patients who find the rehabilitation process more stimulating and less frustrating.

The use of innovative rehabilitation tools is a constantly growing area as it has been observed that standard stroke rehabilitation programs were not effective in achieving an improvement in functional outcomes [29,30]. In this regard, interactive technologies, such as virtual reality (VR), allow the creation of virtual environments in which users are no longer passive subjects but active participants involved in multisensory interactive experiences with objects in virtual space [31]. Thus, it is possible to carry out both cognitive and sensorimotor operations at the same time and provide a multimodal cognitive representation of the virtual interaction [22,32]. Such characteristics are even more pronounced in the most recent and advanced VR solutions such as metaverse and AI technology [28,33,34]. VR and metaverse enable to tap into, in addition to cognition, the affective and social domains thanks to the possibility to embody an avatar that can interact with the space, objects, and others.

Multisensory stimulation refers to an approach that stimulates a person’s tactile, auditory, kinesthetic, and visual modalities [35]. Multisensory technologies (from complex immersive VR, through 2D VR with interactive motion tracking, to PC/tablet) can improve learning efficacy and allow for personalization, since, among other benefits, they allow to select the preferred sensory channel [36]. In support of the efficacy of multisensory stimulation, the literature has shown that sensory stimuli that activate a specific sensory modality can increase a person’s responsiveness to stimuli of other modalities [37,38,39].

Just to make an example of this process, behavioral studies reported that audiovisual tasks, in which subjects had to detect the presence of visual stimuli while ignoring sounds, were able to enhance visual functions, suggesting that spatially and temporally coincidence of sounds and visual stimuli improves visual perception [40].

It is possible to target different sensory modalities by using multiple technological devices; for instance, one could combine headphones, controllers and wearable odor-generating devices to stimulate at the same time the auditory, tactile, and olfactory systems. VR constitutes a good example of this process, given that its stereoscopic displays allow targeting visual–vestibular, proprioceptive, and visual–gustative signals [41,42,43].

In this context, we define multisensory technology as a technological device that involves multiple senses during the interaction. Usually, when interacting with PCs and smartphones, at least two sensory modalities (e.g., vision and motor output) are involved, whereas more advanced technologies such as VR allow multisensory stimulation that involves even three senses (e.g., vision, motor, and auditory systems). Thus, it is possible to differentiate between multisensory technologies and standard PC apparatus looking at the number of sensory modalities involved. Remarkably, a multimodal stimulation approach is closer to the normal perception of reality, which seems to represent a suitable tool for cognitive rehabilitation programs [44]. A systematic review on stroke confirmed this idea [45], as multisensory stimulation programs involving three sensory modalities had a greater positive impact on patients’ cognitive deficits than unimodal and bimodal stimulations.

In light of all these considerations, the purpose of this systematic review is to investigate whether multisensory technologies can be effectively used in the treatment of cognitive deficits in post-stroke patients and, through the analysis of the existing literature, to understand whether this rehabilitation tool could be a valid alternative to conventional treatments. To reach this aim, we included studies that focused on the efficacy of rehabilitation training using multisensory technology in post-stroke patients.

## 2. Materials and Methods

A systematic review of the scientific literature was conducted to find studies reporting the use of multisensory technology for cognitive rehabilitation in post-stroke patients. The research protocol was prospectively registered in the Prospero database (registration number: CRD42021214411) in January 2021, and it is reported here following the Preferred Reporting Items for Systematic Reviews and Meta-Analysis (PRISMA) guidelines [46].

### 2.1. Data Sources and Search Strategy

A systematic electronic search was conducted on 7 July 2020, in the following da-tabases: the Cochrane Central Register of Controlled Trials (CENTRAL), PubMed, and PsycINFO.

Cochrane and Pubmed were chosen as they are the main collection of evidence in healthcare, whereas PsychINFO is the best resource for scientific research results in psychology and related fields and disciplines.

A further systematic electronic search with the same databases was conducted on 17 January 2022 to check whether new studies could be included. No studies were added.

The search and subsequent data extraction were performed independently in each database named above, using specific search strings:

- (Stroke) AND (“multisensory” OR “sensor” OR “sensory feedback” OR “haptic” OR “wearable” OR “brain–machine interface” OR “robotic” OR “computerized training” OR “virtual reality” OR “computer-based” OR “augmented reality” OR “mixed reality” OR “video 180” OR “video 360”)
- (Technology) AND (“cerebrovascular disorders” OR “post-stroke” OR “brain injury” OR “intracranial aneurysm” OR “hemorrhage” OR “intracranial embolism and thrombosis” OR “brain ischemia” OR “ischemia”)
- (“Stroke” OR “cerebrovascular disorders” OR “post-stroke” OR “brain injury” OR “intracranial aneurysm” OR “hemorrhage” OR “intracranial embolism and thrombosis” OR “brain ischemia” OR “ischemia”) AND (“technology” OR “multisensory” OR “sensor” OR “sensory feedback” OR “haptic” OR “wearable” OR “brain–machine interface” OR “robotic” OR “computerized training” OR “virtual reality” OR “computer-based” OR “augmented reality” OR “mixed reality” OR “video 180” OR “video 360”)

In addition, the terms were also combined with the Cochrane Highly Sensitive Search Strategy to identify randomized trials in MEDLINE: sensitivity-maximizing version (2008 revision); PubMed format. The “technology” keyword was chosen instead of “multisensory technology” because there is no pre-existing definition.

Two reviewers (A.P. and F.B.) independently examined all nonduplicate titles and abstracts, searching for eligible articles. The same reviewers retrieved and analyzed the full text for all relevant articles, resolving disagreements by consensus. Another author (D.D.L.) was designated as the third reviewer to arbitrate potential discrepancies unresolved.

First, the complete list of the extracted articles was imported into EndNote to remove duplicates, and then it was imported into Rayyan to check the title and the abstract.

### 2.2. Study Selection and Inclusion Criteria

This review aimed to evaluate the efficacy of multisensory technology in cognitive rehabilitation in post-stroke patients; therefore, “paper and pencil” training and all technologies involving less than three senses were excluded.

Specific inclusion criteria were as follows: stroke diagnosis, adult post-stroke participants (>18 years old), use of multisensory technology for cognitive rehabilitation, RCT studies (control group, blinded group, and random assignment), human subjects’ studies, and English language.

#### Types of Interventions

We included all trials reporting multisensory technology training alone or in combination with another rehabilitation compared with a control group (conventional rehabilitation or another intervention approach with technology).

### 2.3. Data Extraction

The reviewers independently extracted the following data: authors of the study, sample, tools used for cognitive assessment, task, intervention technology, the senses mainly involved in the treatment, and the primary outcomes. Data are available in Table 1.

### 2.4. Risk of Bias Assessment

The methodological quality of the included studies was assessed independently by the two reviewers (A.P. and F.B.) according to the Cochrane risk of bias assessment tool (RoB2 [57]). There were no disagreements. Data are available in Table 2.

Table 2 shows the results for the risk of bias assessment. Most studies showed a low risk of bias across multiple dimensions.

Regarding the risk of bias arising from the randomization process, only Kim et al. [53] showed some concerns because of a difference in the neglect severity between the control and the experimental groups when looking at the standard deviations, which might have influenced the outcome.

Concerning the risk of bias in the selection of the reported result, Maier et al. [54] and Yip at al. [56] were judged high and with some concerns of risk of bias, respectively. Specifically, Maier et al. [54], during the post hoc analysis to compare the baseline scores with those obtained after treatment and at the follow-up, performed a complete case analysis, and the last observation was carried forward analysis to deal with missing data. Significant results were accepted only if confirmed by both analyses. Yip at al. [56] was judged negatively because, when participants failed training levels, they were still forced to move to the next level of difficulty with a possible decrease in self-efficacy.

## 3. Results

Of 12046 non-duplicate studies, 11,852 did not meet the preliminary inclusion criteria; specifically, they did not investigate cognitive rehabilitation with multisensory technology. The full texts of 187 retrieved articles were then analyzed for the specific inclusion criteria. Of these 187 studies, only 10 used multisensory technology to improve cognitive functions in post-stroke patients. Several studies were omitted according to the exclusion criteria: (a) non-RCT studies or other wrong publication types (such as conference posters, book chapters, case reports, theses, meta-analyses); (b) studies that used no multisensory technology or technology in general; (c) studies which did not test cognitive rehabilitation.

**Figure 1:**
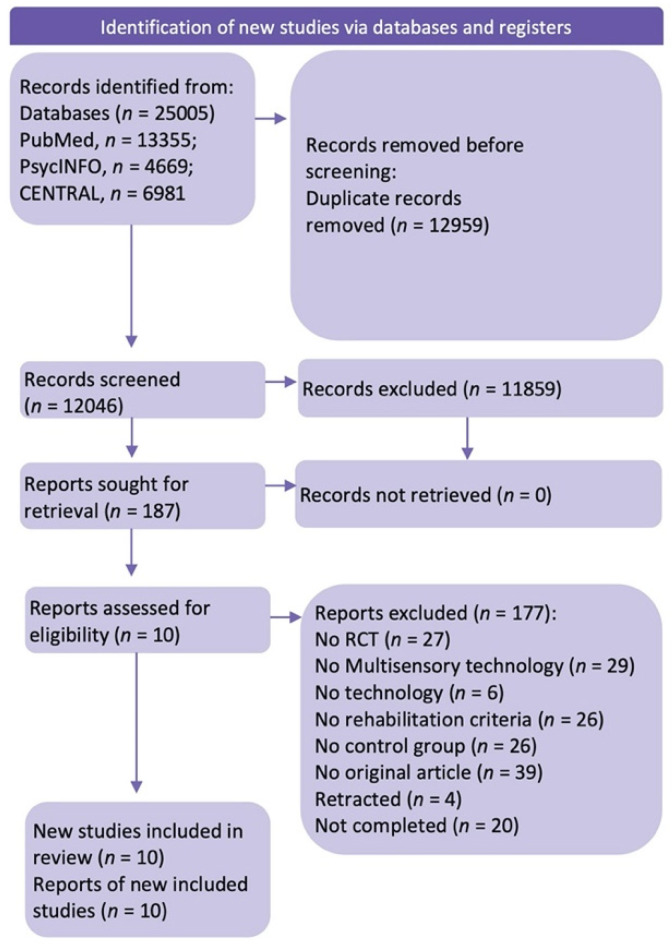
Flowchart of the systematic review (Page, M.J. et al. (2021) [46]).

In the end, 10 papers were suitable for the systematic review (the flowchart of the search strategy is available in Figure 1).

### 3.1. Study Characteristics

Table 1 presents the characteristics of the papers included in this review. Seven out of ten of the selected studies were based on training employing semi-immersive VR technology [48,49,50,53,54,55,56]. Two studies used a computer-based program with an interactive patient–computer interface [51,52], and one of these proposed simulator-based training thanks to an interactive driving scenario [47].

When analyzing the multisensory dimension, it was observed that half of the articles used visual, auditory, and tactile senses [47,49,50,55,56], while the other half employed visual, auditory, and proprioceptive/vestibular systems [48,51,52,53,54].

The clinical sample was evaluated both before and after the treatment to test the rehabilitation efficacy; eight of them included a follow-up assessment [47,48,49,50,51,52,54,55].

### 3.2. Type of Technology Used in Post-Stroke Rehabilitation Interventions

The first objectives of this review were to identify and classify multisensory technology programs used in post-stroke rehabilitation. Where available, project details are described.

We divided the results according to the technology involved, namely, multisensory technology with motion tracking and multisensory technology without motion tracking.

#### 3.2.1. Cognitive Rehabilitation Using Motion-Tracking Multisensory Technology

This type of multisensory technology is a system for monitoring the movement of the body and its specific parts, which allows patients to move and/or act within the virtual space, providing real-time feedback on their movements. Of the five articles that fell into our category, three [48,53,54] proposed a rehabilitation based on VR (non-immersive), while the other two [51,52] developed a PC/screen patient interface.

Analyzing the studies that adopted a rehabilitation program using VR, we noticed that De Luca and colleagues [48] evaluated the effects of VR-based training through the use of an interactive and semi-immersive program for cognitive and motor function recovery. The program used is the BTs-Nirvana, which is based on optoelectronic infrared sensors connected to a projector or maxi screen placed behind the patient. In addition, an infrared camera analyzed the movements of the participants and reproduced them inside the environment. Thus, the rehabilitation exercises allowed engaging patients’ perceptual–cognitive abilities thanks to audiovisual stimuli and the corresponding visual–motor feedback. Several exercises were selected for the rehabilitation to target attention, memory (both verbal and visuospatial), spatial cognition, ocular–manual coordination, gnosis, problem-solving, executive function, and constructive praxis.

In particular, for the executive and visuospatial domains, exercises asked patients to perform ideo-motor sequences, moving or manipulating specific objects (such as balls, butterflies, or flowers) in different directions; for the attention domain, the patient selected some elements, virtual targets, in a given time, producing a visual change in the environment with positive feedback if the target is reached and negative if the target is missed and, therefore, no longer visible on the screen; lastly, for the memory domain, patients were observed while interacting with elements in the virtual environment, both in an immediate time and in a recall time, and they were later asked to remember the position and their names. Instead, at the same time, the control group received standard cognitive training rehabilitation.

Maier and colleagues [54] proposed an adaptive conjunctive cognitive training program (ACCT), which, through the use of semi-immersive VR, was aimed at improving selective, divided, and sustained attention, inhibition, dual-tasking, and spatial awareness. Specifically, the technology used for this type of training consisted of a desktop, an eye tracker, and a Microsoft Kinect with two wristbands worn by the patient for movement detection. Using this system, it was possible to move the virtual arms by detecting the patient’s arms movements on the support surface. Patients assigned to the experimental condition were administered three different training scenarios that proposed multidomain exercises. The first one, called “complex spheroids”, was aimed at rehabilitating attention and memory through a game in which patients had to intercept colored spheres following a predefined sequence indicated at the corner of the screen. The second task, called “star constellations”, asked the patient to replicate a predefined sequence of bright stars arranged in constellations, and it was designed to rehabilitate working memory, spatial memory, and spatial attention. To increase difficulty, the number of stars and the delay period for the recall were manipulated. Lastly, the third task, “quality controller”, engaged the patient in two spatially distributed tasks simultaneously. The patient was asked to remove the donuts from the fryer at the end of the cooking time while identifying the defective cakes. The control group received a set of standard cognitive tasks to complete at home.

Kim and colleagues [53] in their study evaluated the efficacy of a rehabilitation therapy based on VR for patients with unilateral spatial neglect. The experimental setup consisted of a VR system (IREX), a monitor, a video camera, and gloves with sensors worn by the patient. The position and movements of the hands were transmitted and projected into the VR environment. The experimental training consisted of three exercises: “bird and ball” in which patients had to touch flying balls with their hand to turn them into birds, “coconut” in which patients had to recover the coconuts that fell from the tree, and “container”, in which patients had to move a box from one conveyor belt to the other placed on the other sides of the avatar. All the subjects were in a wheelchair because they could not be trained while standing. Instead, the control group received conventional rehabilitation programs such as visual tracking, reading, writing, drawing, copying, and puzzles. In addition, both groups received physical and occupational therapies.

Regarding the studies that used technology based on a patient–PC interface, Kang and colleagues [51] applied motion-tracking technology to a computer-based rehabilitation program for visual perception training to assess its efficacy and applicability. Using the CAMSHIFT algorithm, they developed a tracking technology with an interactive patient–computer interface for training visual perception. Thanks to a computer camera, the software was able to detect and monitor the movements of the patient’s arm and hand and reproduce them on the screen to perform various tasks. To improve visual function, 12 activities were developed and classified into four main domains: visual reactions, visual differential reactions, visual tracking and targeting, and spatial and motor visual challenges. Patients had to use their arms and hands to interact with the environment to reach some elements that appear on the screen, and then they were asked to remember and reproduce a path by selecting certain target elements and following the movement of a moving object by performing fine and precise gestures. Meanwhile, the control group was rehabilitated through the Foundation and Visuospatial sections of PSS CogRehab, which was an eight-module program for the rehabilitation of four domains of cognitive functions: foundations, memory, visuospatial, and problem-solving.

In the same way, Kannan and colleagues [52] evaluated the efficacy of cognitive and motor exergame training for the improvement of both balance control (volitional and reactive) and cognitive, executive, and attentive functions. The exergame was Wii Fit from Nintendo production, which is a video game that uses a balance board (i.e., a balance platform designed to allow users to perceive the symmetry of body weight distribution). In conjunction with balance games, for cognitive rehabilitation, cognitive exercises were proposed to improve semantic memory, abstract memory, working memory, attention, and verbal fluency; for this last purpose, they used tasks such as category fluency, word list generation, digit recall, mental arithmetic, analogies, and letter repetition. As for the control group, conventional training was administered including a series of personalized balance training exercises for 90 min.

#### 3.2.2. Cognitive Rehabilitation Using No Motion-Tracking Multisensory Technology

This type of multisensory technology uses devices such as a joystick, keyboard, touch table, and 3D objects to interact in the virtual environment. Among the considered studies, all but one [47] chose rehabilitation through non-immersive VR.

Rogers and colleagues [55] investigated the efficacy of Elements, an interactive VR system for cognitive and motor functions rehabilitation. The rehabilitation program consisted of seven increasingly structured tasks, in which the participant had to perform a series of assignments interacting with a 42 inch touchscreen table; the tasks required manipulating four three-dimensional objects of different shapes (circle, pentagon, triangle, and rectangle). More precisely, the system provided two types of user interactions: goal-oriented and exploratory, which focused on speed and accuracy to promote motor learning and cognitive control. Goal-directed activities involved moving, with a single hand, lifting or sliding, and positioning a circular object on selected targets. The first task, “bases”, had the circular objective targets positioned in a fixed order, east, north, west, and south, which were activated through the use of an illuminated edge; the “random bases” task had the same configuration as the first one but with the difference that the targets are highlighted in random order; the “chase” task, from an initial white screen, a target circle appeared in one of the positions to be reached; the “go/no-go” task, with the same positions as Task 3, added distractor targets, a pentagon, a triangle, and a rectangle, and required participants to place the object only on the circular targets.

Exploration activities required participants to explore the virtual environment, creating various shapes and sounds through movement. Specifically, the “mixer task” consisted of nine circles in a 3 × 3 grid, in which the movement of the circular object activated the sound and the animation of the rotating edge; “the squiggles” task presented a blank display on which participants could draw lines and shapes by sliding any of the four different hand-held objects across the screen, and as each object was moved an animation of the track was drawn along its path producing a musical tone; the “swarm” task encouraged two-hand control to explore the audiovisual relationships between all four objects held in the hand. In this way, multiple-colored shapes were positioned on the screen that slowly gravitates and swarms around the base of each object held in the hand. During activities, increased auditory and visual feedback was presented in real time, reinforcing movement-related attributes such as speed, trajectory, and endpoint contact. The experimental group received 12 VR sessions with Elements. The control group, on the other hand, received only the standard treatment which was customized based on the collaborative care planning objectives established by the patient and the treating team. The most common tasks focused on a series of movement exercises, muscle strengthening, coordination, and retraining of daily life skills such as eating, going to the bathroom, getting dressed, and making transfers.

Faria and colleagues [49,50] proposed a multisensory cognitive rehabilitation based on semi-immersive VR. Reh@City is a software which presents a three-dimensional reconstruction of a city. It can be installed on a PC and the user works on a table, in front of an LCD monitor, moving a handle on the table surface with the paretic to interact with the virtual content, intending to improve cognitive functions. The movements of the user’s upper limbs were captured through augmented reality tracking software using the “PlayStation Eye” camera and then mapped into the movements of a virtual arm (in indoor activities) or as directions of movement (when navigating outdoors) in the environment. This technology enables an integrated and personalized cognitive rehabilitation process, targeting different cognitive domains such as memory, attention, executive functions, and visual–spatial skills in an environmentally sound approach. Through the immersive environment, subjects find themselves interacting in daily life environments. Specifically, the focus is to rehabilitate people to carry out some of their daily activities by him or themselves. Some of the most common activities proposed are going to the supermarket, pharmacy, or post office to buy items or pick up products; selecting the receipts to pay; finding routes to reach VR shops; storing verbal information from a newspaper for later “true or false” recall. The control group was involved in standard cognitive treatment.

In a very similar way, Yip and colleagues [56] developed the virtual reality-based prospective memory (VRPM) training, which aims to rehabilitate perspective memory through a cognitive rehabilitation program based on a non-immersive VR setting involving sight, hearing, and touch. The VRPM program was developed in 3D layout, and participants could choose to use the joystick or keyboard control as an input device. The VR scenario was a minimarket where the subjects had to make purchases (shopping). This is because patients could experience a real-life environment in which prospective memory could have educational content. More specifically, the VRPM program consisted of three different pieces of training aimed at improving prospective memory, retrospective memory, and inhibition component. Regarding prospective memory, tasks based on events were developed such as the purchase of products at discounted prices, or time-based activities, such as removing food from the microwave after a few minutes, and again, tasks (ongoing) such as purchasing items by following a given shopping list. Afterward, for the retrospective memory, tasks were used in which the user had to memorize a shopping list consisting of four items and then, a second time, choose the correct items (i.e., those stored) from a list of eight. Lastly, for the inhibitory components, three different items were individually presented to the subjects on a screen, representing a product with a “special price” tag, a product with a “new” tag, and a product without a tag. The subjects were asked to press the spacebar whenever a product with a “special price” tag appeared on the screen. Meanwhile, during the treatment phase, the control group had regular reading and table games activities.

Lastly, the study by Akinwuntan [47] proposed a different rehabilitation technology. Using traditional therapy combined with training based on an interactive driving simulator, they sought to improve general driving skills by focusing on the rehabilitation of the visual attention and processing speed domains. The simulator, developed by STISIM Drive System (version 1.03; Systems Technology Inc, Hawthorne, California), includes a semi-immersive screen featuring driving scenarios and a full-size Ford Fiesta car with automatic transmission and with all parts of original mechanics. In addition, adaptive aids are provided including the accelerator pedal on the left side, the turn signal stick on the right side and the steering. Ten driving scenarios with 5 km each were created. Four of these were used for the rehabilitation of divided attention, and it was simulated with four different sequences of presentations: a lonely street without houses, pedestrians, or other vehicles. The remaining six scenarios proposed the simulation of normal daily traffic and were used for the rehabilitation of selective attention. All the scenarios featured two red diamonds on the screen placed respectively centrally on the right and left of the screen, moving randomly. The patient was instructed to react as quickly as possible to the switch of diamonds. These changed their shape at regular intervals, one at a time, into a triangle, pointing to the right or left, or into a horn shape. Patients had to make a right turn when the right-facing triangle appeared, a left turn for the left-hand triangle, and a ring when the horn appeared. Instead, the control group received 15 h of cognitive skills training using the Tantrix Complex puzzle for reasoning and memory, the Take it Easy puzzle for perception, planning, and decision making, and the puzzle of Rush Hour for executive skills.

### 3.3. Efficacy of Technology-Based Rehabilitation Programs

The second aim of the present review was to investigate the efficacy of these multisensory technologies for cognitive rehabilitation in post-stroke patients.

#### 3.3.1. Motion-Tracking Multisensory Technology

For the motion-tracking multisensory technology studies, it is possible to observe that all the studies [48,51,52,53,54] reported a post-intervention improvement. Two of these [48,51] showed similar results, observing the improvements as independent from the type of rehabilitation treatment administered; conversely, Kannan and colleagues [52] and Maier and colleagues [54] found that the experimental group achieved significantly better improvements compared to the control group. Lastly, the study by Kim [53], despite reporting significant improvements for both groups, showed that this improvement was not maintained at follow-up.

De Luca and colleagues [48] found that, at the end of the rehabilitative treatment (T1) the experimental group presented greater improvements compared to the control group both in the attentive matrices (MA), visuospatial and attention domains evaluated with Montreal Cognitive Assessment (MoCA), verbal memory, and constructive abilities, and in motor operation measures as the trunk control test and the upper limb motricity index scale (MI). In addition, it was found that these improvements persisted 1 month after the end of treatment (T2) only in the experimental group.

Similar results were observed by Kang and colleagues [51]; findings showed that both experimental and control groups reported a significant improvement after the Mini-Mental State Examination (MMSE), as well as in the visual perception domain. However, the difference between the two groups seemed to be related to the level of interest in the rehabilitation program measured with a self-report scale. More specifically, the experimental group reported a significantly greater interest than the control group.

The results by Kannan and colleagues [52] showed that the experimental group improved both cognitive and motor functions, as opposed to the control group, which experienced only motor progresses. Referring to cognitive activity, only the experimental group showed a significant pre–post difference in terms of accuracy after the cognitive training. Similarly, Maier and colleagues [54] reported significant improvements in the experimental group. Going more into detail, the averaged standardized composite score (ASCS) was used as the primary outcome; this was obtained by combining the standardized average scores of four cognitive domains: attention, memory, executive functions, and spatial awareness. The measures of each domain were collected through the administration of different test batteries that led to a joint score representing the domain itself in the final ASCS construct. The results found significant changes in the ASCS construct for the experimental group in the domain of attention, spatial awareness, and generalized cognitive functioning. Specifically, it was observed that, with regard to the domain of attention, the post hoc analysis revealed significantly higher scores at the follow-up (3 months from the end of the intervention) compared to the baseline, while, for the domains of spatial awareness and generalized cognitive functioning, post hoc analysis revealed significantly higher scores both at the end of treatment and at follow-up than at baseline. For the control group, no significant change over time was found. Lastly, for both groups, no significant improvements in the executive function domain were found.

In conclusion, the results by Kim and colleagues [53] showed significant post-treatment improvements in all tests administered (i.e., the star cancellation test, line bisection test, CBS, and Korean version of the modified Barthel index) for both groups, suggesting an improvement in the degree of negligence. Precisely greater improvements were recorded in the experimental group in the star cancellation test and the K-CBS scores after treatment compared to the control group. However, 3 weeks after the end of treatment, no significant differences were maintained in the line bisection test and K-MBI in both groups. There has been no improvement in the activities of daily living.

#### 3.3.2. No Motion-Tracking Multisensory Technology

As for the no motion-tracking multisensory technology studies, it can be observed that all but one (Akinwuntan [47]) reported significantly greater improvements for the experimental group compared to the control group.

In the study by Faria and colleagues [49], the within-group analysis showed significant improvements in global cognitive functioning, measured through the Addenbrooke’s cognitive examination (ACE) and the MMSE, especially in the attention and memory domains and in visuospatial ability only in the experimental group. The control group showed improvements only in self-reported memory and social participation. Executive functions were evaluated through the picture arrangement test (from WAIS-III), which showed a significant improvement at the end of the treatment only within the experimental group. The between-group analysis showed significant improvements in global cognitive functioning, executive functions, and attention, measured with the ACE, in the experimental group compared to conventional therapy. However, the Trail Making Test (TMT) did not improve. Lastly, for the measurement of general health status (SIS) both the experimental group and the control group registered significant improvements. For the experimental group, improvements were detected in the domains of strength, memory, mobility, emotion, social participation, and overall recovery, while, for the control group, improvements were detected in those of mobility, memory, and social participation.

As confirmation of these first data, in a second experiment also conducted by Faria and colleagues [50], the results were similar; significant improvements were shown only for the experimental group in the domains of general cognitive functioning, visuospatial ability, executive functions, and attention measured with the MoCA, while, for the control group, the only significant change was observed in the orientation subdomain. Regarding the data collected by the TMT for attention analysis, it was shown that only the experimental group showed a significant improvement in reducing the time for the completion of the test before and after the intervention. The memory domain, in post-treatment, analyzed through the Wechsler memory Scale—third edition (WAIS-III) found significant improvements within the experimental group for retention and recognition, while improvements in the control group are only significant for the retention score. Regarding the executive functions, the experimental group reported significant improvements in the digit symbol coding test, while the control group did so in the symbol search at the follow-up. The language, analyzed through WAIS-III, showed that only the experimental group reported improvements in vocabulary evaluation at follow-up.

The results of Rogers and colleagues [55] show that both groups reported a significant improvement in motor, cognitive, and functional status. In particular, the experimental group showed a significantly greater improvement in motor function (box and blocks task, BBT) in the hand most affected by stroke, in functioning in everyday life situations, and in all measures of cognitive function (MoCA and cog-state tasks) compared to the control group between pre and post treatment. Furthermore, the improvements obtained by the experimental group were persistent during the 1-month follow-up. Other group differences emerged from the self-report measures on the functional performance of the neurobehavioral functioning inventory (NFI); the patients in the experimental group, at the end of the intervention, perceived a recovery approximately 2–3 times higher than that of the control group.

In the study of Yip and colleagues [56], within-group analysis revealed that the experimental group showed significant improvements in most of the tests. These included the immediate recall of prospective memory (PM) tasks, the performance of both event-based and time-based PM tasks, the performance of ongoing tasks, and the number of time checks. The real-life behavioral PM also showed a significant improvement in time-based and event-based activities, but not in ongoing activities. For other standardized evaluations about the cognitive profile of participants, only total scores of the Cambridge Prospective Memory Test (CAMPROMPT; a test investigating prospective memory), the Frontal Assessment Battery (FAB) battery that measured frontal lobe skills and executive functions, and the Word Fluency Test—Chinese Version (WFT-testCV), measuring the fluency RBAL, showed a significant improvement. At the same time, no significant difference was found in any outcome measure in the control group. The results showed a significant difference between the experimental group and the control group in the event-based task scores in real-life behavioral PM tests, in the FAB battery, in the WFT-CV test, and in test 1 of the color trails test (CTT). In conclusion, the findings suggest significantly better changes in both VR-based and real-life perspective memory outcome measures and related cognitive attributes such as frontal lobe functions and semantic fluency.

Lastly, in the study of Akinwuntan and colleagues [47], the experimental and control groups significantly improved the speed of processing and divided and selective attention. However, the simulator-based driving program did not rehabilitate patients better than a non-computer-based cognitive training program.

## 4. Discussion

This review aimed to identify the different multisensory technologies used in the cognitive rehabilitation of post-stroke patients to determine their potential efficacy and usefulness in the clinical setting. The primary goal was to understand whether technological innovation could lead to a real contribution to the field of cognitive rehabilitation.

Looking at these preliminary results, it is possible to identify some areas of interest to better investigate and consider in future research. Specifically, multisensory technology and usual treatment seem to be both effective, however, the former seems to be more suitable for specific domains such as attention, visuospatial processing, memory, and global cognition. Secondly, multisensory technologies could be categorized into motion-tracking and without motion-tracking devices, and this difference could affect the cognitive rehabilitation outcomes, where the former seemed to be more effective than standard treatments, whereas the latter was at least comparable to the usual care. Thirdly, current trials did not use tasks or MSI tests to assess the impact of multisensory technology on multisensory processing or a multimodal evaluation. Fourthly, available studies mainly focused on sub-acute and chronic conditions; thus, it could be interesting to extend the investigation for example to acute ones. Lastly, further research should try to control for methodological biases of clinical research (e.g., blinding and a priori sample size).

Regarding the cognitive functions investigated, the studies showed a significant improvement mainly in attention, memory, executive functions, visuospatial skills, and global cognitive functioning. Both motion-tracking and no motion-tracking multisensory technology proved, in some cases, to be effective for cognitive improvement compared to the standard treatments [48,50,52,54,55,56,58]. However, it cannot be ignored that, in other cases, the improvement occurred in both groups regardless of the type of treatment, which is proof of the non-electivity of the multisensory technology [47,51,53,54]. Overall, the analysis and evaluation of the results reported by each selected study seemed to suggest the ability of multisensory technology to produce an improvement at the level of cognitive functions equal to, and sometimes greater than, conventional treatment. Furthermore, some studies showed that these improvements were also maintained at follow-up.

The analysis of the selected studies suggested a macro-classification of the multisensory technology used; specifically, it was possible to identify a motion-tracking technology and a non-motion-tracking technology.

In the first case, the movement in virtual space mirrors the patient’s body movement, meaning that the subjects, by moving their limbs (almost always upper ones), were able to move the avatar in the virtual setting. Instead, in the case of non-motion-tracking technology, three-dimensional objects or tools were used as intermediaries, meaning that they allowed patients to act spatially in an interactive way without a kinetic controller.

From the literature analysis, it seems that multisensory motion-tracking technology was successfully applied in tasks for divided and selective attention and visuospatial skills. Such improvements, in some cases, were also maintained 1 and 3 months after the treatment ended [48,54,58]. However, in our review, better outcomes compared to the usual cognitive rehabilitation were reported in the non-motion-tracking system studies.

Regarding cognitive assessment, the studies often used only tests of global cognitive functioning from which an improvement in specific cognitive functions is deduced by analyzing the various subtests. However, this could pose a possible assessment bias as it would be more effective to combine broad assessment instruments such as MMSE and MoCA with more specialized diagnostic tests for the cognitive functions that need to be investigated [59,60].

Another evident aspect was the presence of a multisensory treatment, which is often not accompanied by a multidimensional and MSI evaluation. Indeed, most of the considered studies investigated both the cognitive and the motor dimensions, even though the relationship between the two was not investigated and they were treated as distinct and separate entities. In addition, crucial phenomena in body, environment, and body in environment perception—such as proprioception, interception, and body representation—were not considered.

Furthermore, the studies included different post-stroke time windows; in half of the patients, the stroke occurred between 3 to 6 months [48,49,50,56], while, in some of the studies, it dated back to a period between 6 and 10 months [52,54], and, in other cases, the critical event ranged from 1 month up to 2 months before [51,53,55]. The most promising results appear to have been obtained in studies with a post-stroke time window between 3 and 6 months.

Some limitations should be noted concerning the conclusions of this review. Firstly, the selected studies were included for a qualitative analysis; thus, there were limited data concerning the quantitative efficacy of multisensory technologies in enhancing cognitive functioning in post-stroke patients. Linked to this, most of the studies used a small sample size, without estimating a priori the number of participants needed to reach a considerable effect size. Secondly, this review considered mainly registered RCTs, since they represent the gold standard in the evaluation of interventions in healthcare, and other types of reports were excluded due to methodological concerns; this attempt to collect results with a certain degree of quality and reliability and the limited number of studies in the field made the number of studies relatively small. Lastly, results should be analyzed carefully, given the heterogeneity of participants and interventions (e.g., the task proposed and the software used) which did not allow making direct comparisons.

Thus, even though results to date suggest multisensory technologies as a suitable alternative to paper-and-pencil procedures in the context of cognitive rehabilitation in post-stroke patients, more research is needed to drive generalizable conclusions.

## 5. Conclusions and Future Directions

Future research should try to address the limitations underlined in the included studies, which implies (1) improving technological solutions (e.g., immersive VR) of both motion and non-motion-tracking technologies, (2) including comprehensive neuropsychological and MSI assessment, and (3) ameliorating clinical (e.g., acute phase rehabilitation, neuroimaging information) and methodological aspects (e.g., bias).

Regarding the technological aspect, multisensory technology seems to represent a potentially valid tool mainly in the treatment of attention, memory, visuospatial skills, and global cognition with benefits at least comparable to and sometimes better than conventional treatment. Certainly, it is not currently possible to think about a predominance of this type of procedure given the lack of adequate rehabilitation protocols. However, available preliminary data suggest that it is worth going into more detail to further stimulate patients in various phases of the stroke where MSI and cognitive deficits could arise. The recent advances in theory [22,61], technology [62], and the field of VR [33] could improve and move this type of technological clinical application to the next generation of multisensory technologies.

Our way of perceiving space, body, and others, as well as the relationship of our mind with the environment, is conveyed by a multisensory experience that is constantly being updated and adapted. We believe that a technology that can ‘recreate’ a similarly complex (multisensory) reality could be a key turning point for the rehabilitation of cognitive deficits and might be applied in several different conditions that imply alterations of bodily perception [63,64,65,66]. Furthermore, considering the mind as embodied and in relation with the world, both internal and external, a ‘simulated multisensory experience’ could stimulate a re-enactment of the internal model through bottom-up mechanisms. In particular, the metaverse is a transformative technology, capable of modifying what people think reality is through the simulation of virtual multisensory experiences.

In conclusion, the current state of the art seems to suggest that the application of multisensory technologies for post-stroke cognitive deficits is a promising field to address cognitive impairments in different domains such as attention, memory, executive functions, visuospatial skills, and global cognitive functioning. Indeed, this review presented preliminary studies that encourage further exploration of this research area to develop clear clinical, technical, and research guidelines that would allow using multisensory technologies in ordinary clinical practice for cognitive rehabilitation programs.

## Figures and Tables

**Table 1:** Study characteristics.

Author(s) and Year	Sample	Assessment	Task	Cognitive Function	Technology	Multisensory	Primary Outcome
Akinwuntan et al. 2010 [47]	EG: 33 CG: 36 Side of lesion: both Time post-stroke: not specified	UFOV test pretraining at 6 to 9 weeks, and follow-up at 3 months and 6 months post-stroke EG: 15 h of training over 5 weeks at 1 h per session, 3 sessions per week with following subtests: - divided attention - selective attention - speed of processing	The participants in the experimental group received specific training to improve their visual attention and speed of processing skills. The task was composed of 10 scenarios.	Visual attention skills	STISIM Drive System (version 1.03; Systems Technology Inc, Hawthorne, CA, USA)	Three senses: - sight - hearing - touch	Results revealed neither group effects nor significant interaction effects of group with time in the UFOV total score and the 3 subtests.
De Luca et al. 2018 [48]	EG: 6 CG: 6 Side of lesion: not specified Time post-stroke: 3–6 month	Pre–post intervention and follow-up (1 month). Each treatment session (24) lasted 45 min and was repeated three times a week for 8 weeks. Subtests: - MoCA - FIM - In addition: - FAB - Attentive Matrices - Trial Making Test (TMTA; TMTB, and TMTB-A) - TCT and MI	The device created a special setting called a sensory room. The rehabilitation was composed of exercises with audiovisual stimuli and feedback involving the perceptual-cognitive skills of patients, resulting in a motivational training.	Attention, executive, and spatial-visual functions; verbal and spatial–visual memory.	BTS-Nirvana (Garbagnate Milanese, Italy) using an interactive semi-immersive program (I-SIP)	Three senses: - sight - hearing - proprioception	MoCA and MA improved significantly only in the EG at post-treatment. Particularly in visuospatial attention domains, MI and TCT improved more in the EG than the CG did at T1.
Faria et al. 2016 [49]	EG: 9 CG: 9 Side of lesion: both Time post-stroke (months): EG, 7 (4–49); CG, 4 (3–11.5)	All patients were evaluated pre and post treatment 4 to 6 weeks; 12 interventions, of 20 min each session, distributed from 4 to 6 weeks. Subtests: - ACE - Trail Making Test A and B - Test picture arrangement to WAIS III - Stroke Impact Scale (SIS 3.0)	See Faria 2020 [50]	Global functioning, memory, attention, executive functions.	Reh@City v2.0	Three senses: - sight - hearing - touch	In EG, there were significantly greater improvements in global cognitive functioning ACE and MMSE, attention, and executive functions than in conventional therapy.
Faria et al. 2020 [50]	EG: 17 CG: 19 Side of lesion: both Time post-stroke, at least 6 months (months): EG, 45.93; CG, 21.33	Pre and post intervention at 4–6 weeks, and follow-up at 2 months; 12 sessions of treatment. Subtests: - MoCA - Trail Making Test A and B - Verbal Paired Associates to WMS-III - Digital span forward and backward - Digital symbol coding - Wechsler Adult Intelligence Scale III (WAIS)	Reh@city is a virtual reality simulation of a city where patients are required to solve cognitive tasks through familiar activities of daily living (ADL) in a variety of common places: buying food in a supermarket, picking up a package in the post office, paying the electricity at the bank ATM, etc. These places display billboards and real products of actual spaces and trademarks to help the patient relate the VR task to the real world. In addition, patients are also required to use their paretic arm to solve the tasks.	Global functioning, memory, attention, executive functions, and language.	Reh@City v2.0-based simulation of ADLs; customized handle with a tracking pattern on the surface of the table	Three senses: - sight - hearing - touch	In EG, there were significantly greater improvements in general cognitive functioning, visuospatial ability, executive functions, and attention on the MoCA.
Kang et al. 2009 [51]	EC: 8 CG: 8 Side of lesion: right Time post-stroke (days): EG, 64.3; CG, 58.1 (29.9)	Pretraining and post-training at 4 weeks; a total of 12 sessions (3 per week) for 30 min per session for 4 weeks. Subtests: - MMSE - Motor-Free Visual Perception Test - K-MBI	The program consisted of 12 tasks in four parts designed to improve visual perceptual function: - Task of visual reaction - Task of visual differential - Task of visual tracking and targeting - Task of visual–spatial cognition and moto functions	Global functioning, visual perception.	PSS CogRehab program (Indianapolis, IN, USA); motion-tracking technology based on the CAMSHIFT	Three senses: - sight - hearing - proprioception	The mean motor-free visual perception test score increased significantly in both EG and CG. The Barthel index score increased significantly in both groups, with the EG recording a higher increase. The mean interest scale score was greater in the EG than in the CG.
Kannan et al. 2019 [52]	EG: 10 CG: 10 Side of lesion: both Time post-stroke: greater than 6 months ago	At pre–post test and follow-up at 11 weeks; 6 weeks of training, 10 sessions, 90 min for each session. Subtests: - MoCA - WRAT-4 - BBS - TUG - 6MWT - ABC - SPT + LNS - LOS + LNS - B + Cog-Balance and Cognitive task	Participants played Wii Fit games in conjunction with performing cognitive tasks. Each session was divided into three sub-sessions, with each sub-session comprising four Wii Fit games: Bubble; Table Tilt; Tight Rope Walking; Soccer Heading. The cognitive games were played in conjunction with each of the balance games and these have trained the semantic memory, abstract memory, working memory, attention, and verbal fluency.	Attention, executive functions, WM.	Wii Fit (Nintendo Co, Ltd. Kyoto, Japan), balance board	Three senses: - sight - hearing - proprioception	The EG showed a significant improvement in both motor and cognitive functions in particular areas: - movement velocity while performing the limits of stability (LOS) test under dual-task conditions. - COM state stability and cognitive ability during the dual-task slip-perturbation test. - cognitive function that was improved during dual-task performances under slip-perturbation test. - In the CG, only the motor functions were improved.
Kim et al. 2011 [53]	EG: 12 CG: 12 Side of lesion: right MCA infarction Time post-stroke (days): EG, 25.5; CG, 22.8	Pre-training, post-training; therapy for 30 min a day, 5 days per week for 3 weeks. Subtests: - Star cancellation test - Line bisection test - CBS - K-MBI	The VR training was composed of three programs at a time: “bird and ball”; “coconut”; “container”. In the three programs, patients were told to use nonparetic right hands against the left simulation.	Visual–spatial attention.	IREX system^®^ (Toronto, Ont., Canada) is a VR system consisting of a monitor, a video camera, computer-recognizing gloves, and virtual objects.	Three senses: - sight - haring - proprioception	The EG showed improvements in attention, spatial awareness, and generalized cognitive functioning. No significant change was seen in the executive function and memory domain. For the CG, no significant change over time was found. The EG displayed a lower level of depression than the CG after treatment.
Maier et al. 2020 [54]	EG: 16 CG: 14 Side of lesion: not specified Time post-stroke: more than 6 months after stroke but less than 10 years	At baseline, post-training at 6 weeks and follow-up at 18 weeks; daily training for 6 weeks (30 min each session). Subtests: - ASCS - Corsi f - TMT A - WAIS - Corsi B - MoCA - MMSE - BI - FM-UE	Different VR scenarios for the training of spatial attention and working memory. The patient controls a virtual avatar on a computer screen, with conjunctive cognitive training scenarios which ACCT. The patients are seated at a table, and the three training scenarios are shown on the screen always in the same order. The complex spheroids The star constellations The quality controller	Attention, executive functions, spatial awareness, memory.	VR rehabilitation tool RGS with desktop computer, Microsoft Kinect, two wristbands with reflective markers (worn by the patient), and a Tobii Eye tracker T120.	Three senses: - sight - hearing - touch	The EG showed improvements in attention, spatial awareness, and generalized cognitive functioning. No significant change was seen in the executive function and memory domain. For the CG, no significant change over time was found. The EG displayed a lower level of depression than the CG after treatment.
Rogers et al. 2019 [55]	EG: 10 CG:11 Side of lesion: both Time post-stroke (days): EG, 22.8; CG, 30	At pre–post-treatment at 3 weeks and follow-up at 1 month. EG: 4 weeks of Elements virtual rehabilitation (three weekly 30–40 min sessions) combined with treatment as usual. EG and CG both received 3 h of daily conventional occupational and physiotherapy. Subtests: - BBT - GMLT - SST - NFI	Elements: a series of unimanual goal-directed tasks and audiovisual exploration tasks focus on speed and accuracy to promote motor learning and cognitive control. In particular, the rehabilitation programs consist of 7 tasks, increasingly structured, where the participants have to use four handheld objects (the four “elements” in the shape of a circle, pentagon, triangle, and rectangle) and are engaged with a virtual environment.	Global functioning, executive functions.	Elements The system consists of a large tabletop surface display (42 in), tangible user interfaces, and software for presenting both goal-directed and exploratory virtual environments.	Three senses: - sight - hearing - touch	Both groups showed significant training-related improvement in motor and cognitive functions and functional status. However, the magnitude of effect sizes was substantially larger for the EG compared with the CG. In particular, in pre–post difference scores, the EG showed significantly greater improvement. In moto function (BBT) in the hand most affected by their stroke and on all measures of cognitive function compared with the CG. Furthermore, the improvement shown by the EG as a function of training was maintained at the 1-month follow-up assessment; on difference scores between pre-test and follow-up, the EG performed significantly better than controls on motor, cognitive and functional outcomes.
Yip et al. 2013 [56]	EG: 19 CG: 18 Side of lesion: not specified Time post-stroke (days), at least 3 months post injury: EG, 145.13; CG, 167.53	Pre–post-intervention at 3 weeks and follow-up at 1 month. The program, 12 sessions, was run twice a week and each session lasted about 30 to 45 min. Subtests: - MMSE-CV - TONI-3 - ADI-CV - Virtual reality-based test of everyday - Prospective memory task - Behavior checklist of a prospective memory task in a real environment - CAMPROMT-CV - HKLLT - FAB - WFT-CV - CTT - CIQ-CV - Self-efficacy questionnaire in performing an everyday prospective memory task	Three training components were investigated: Prospective memory: the PM training consisted of event-based tasks (such as shopping for drinks with discounted prices or calling back home when a gift redemption counter was seen), time-based tasks (such as taking the food out of the microwave oven after 5 min), and ongoing tasks (shopping for items on a shopping list that had been given). Retrospective memory: in the RM training component, subjects were required to remember a list of four shopping items. Inhibition training component: participants were required to press the spacebar whenever they saw an item with a special price tag shown on the screen.	Perspective memory, retrospective memory.	VRPM: A virtual reality-based prospective memory training program was developed using a non-immersive form of VR. The 3D layout in the program was designed and built by Maya 8.0 Software called Virtools.	Three senses: - sight - hearing - touch	The results suggest that significantly better changes were seen in both EG and CG PM outcome measures, related to cognitive attributes such as frontal lobe functions and semantic fluency. In particular, the EG showed significant improvements in most of the test items: immediate recall of PM tasks, the performance of both event-based and time-based PM tasks performance of ongoing tasks, and number of time checks; in CG, the PM test showed significant improvement in event-based and time-based tasks but not in ongoing tasks.

EG: experimental group; CG: control group; UFOV: useful field of view; MoCA: Montreal Cognitive Assessment; FIM: functional independence measure; FAM: functional assessment measure; FAB: frontal assessment battery; VR: virtual reality; TMT-A: Trail Making Test part A; TMT-B: Trail Making Test part B; TCT: traditional cognitive training; MI: motricity index; ACE: Addenbrooke’s cognitive examination; WAIS-III: Wechsler Adult Intelligence Scale—third edition; WAIS: Wechsler Adult Intelligence Scale; WMS -III: Wechsler Memory Scale—third edition; ADL: activities of daily living; MMSE: Mini-Mental State Examination; K-MBI: Korean version of the modified Barthel index; BBS: Berg balance scale; TUG: timed up and go test; 6MWT: 6-meter walk test; ABC—activity-specific balance confidence; SPT: slip-perturbation test; LNS: letter–number sequencing; COM: center of mass; WRAT4: Wide Range Achievement Test 4; LOS: limits of stability test; WAIS: Wechsler Adult Intelligence Scale—fourth edition; Corsi B: Corsi Block Tapping Test Backward; ACCT: adaptive conjunctive cognitive training; BI: Barthel index; FM-UE: Fugl–Meyer assessment for the upper extremity; ASCS: averaged standardized composite scores; Corsi F: Corsi Block Tapping Test Forward; RGS: rehabilitation gaming system; RAVLT I: Rey Auditory Verbal Learning Test Immediate; BBT: box and blocks task; GMLT: Groton maze learning task; NFI: Neurobehavioral Functioning Inventory; SST: set shift task; TONI-3: Test of Nonverbal Intelligence—third edition; MMSE-CV: Chinese version Mini-Mental State Examination; VRPM: virtual reality-based prospective memory; CAMPROMT—CV: Cambridge Prospective Memory Test—Chinese Version; HKLLT: Hong Kong list learning test; WFT—CV: word fluency test—Chinese version; CTT: color trails test; CIQ–CV: Chinese version of the Community Integration Questionnaire.

**Table 2:** Risk of bias assessment.

Authors(s) and Year		Domain 1: Risk of Bias Arising from the Randomization Process	Domain 2: Risk of Bias Due to Deviations from the Intended Interventions	Domain 3: Missing Outcome Data	Domain 4: Risk of Bias in the Measurement of the Outcome	Domain 5: Risk of Bias in the Selection of the Reported Result	Overall Risk of Bias
1	Akinwuntan et al. 2019 [47]	Low	Low	Low	Low	Low	Low
2	De Luca et al. 2017 [48]	Low	Low	Low	Low	Low	Low
3	Faria et al. 2016 [49]	Low	Low	Low	Low	Low	Low
4	Faria et al. 2020 [50]	Low	Low	Low	Low	Low	Low
5	Kang et al. 2009 [51]	Low	Low	Low	Low	Low	Low
6	Kannan et al. 2019 [52]	Low	Low	Low	Low	Low	Low
7	Kim et al. 2011 [53]	Some concerns	Low	Low	Low	Low	Some concerns
8	Maier et al. 2020 [54]	Low	Low	Low	Low	High	High
9	Rogers et al. 2019 [55]	Low	Low	Low	Low	Low	Low
10	Yip et al. 2013 [56]	Low	Low	Low	Low	Some concerns	High

Low (low risk of bias); high (high risk of bias).

## Data Availability

Not applicable.
